# Urinary Biomarkers of Strawberry and Blueberry Intake

**DOI:** 10.3390/metabo14090505

**Published:** 2024-09-18

**Authors:** Ya Gao, Rebecca Finlay, Xiaofei Yin, Lorraine Brennan

**Affiliations:** 1 Institute of Food and Health, UCD School of Agriculture and Food Science, University College Dublin, Belfield, D04 V1W8 Dublin, Ireland; ya.gao@ucd.ie (Y.G.); rebecca.finlay.1@ucdconnect.ie (R.F.); xiaofei.yin@ucd.ie (X.Y.); 2 UCD Conway Institute, University College Dublin, Belfield, D04 V1W8 Dublin, Ireland

**Keywords:** biomarkers, food intake, metabolomics, strawberries and blueberries

## Abstract

**Introduction** There is increasing interest in food biomarkers to address the shortcomings of self-reported dietary assessments. Berries are regarded as important fruits worldwide; however, there are no well-validated biomarkers of berry intake. Thus, the objective of this study is to identify urinary biomarkers of berry intake. **Methods** For the discovery study, participants consumed 192 g strawberries with 150 g blueberries, and urine samples were collected at 2, 4, 6, and 24 h post-consumption. A dose–response study was performed, whereby participants consumed three portions (78 g, 278 g, and 428 g) of mixed strawberries and blueberries. The urine samples were profiled by an untargeted LC-MS metabolomics approach in the positive and negative modes. **Results** Statistical analysis of the data revealed that 39 features in the negative mode and 15 in the positive mode significantly increased between fasting and 4 h following mixed berry intake. Following the analysis of the dose–response data, 21 biomarkers showed overall significance across the portions of berry intake. Identification of the biomarkers was performed using fragmentation matches in the METLIN, HMDB, and MoNA databases and in published papers, confirmed where possible with authentic standards. **Conclusions** The ability of the panel of biomarkers to assess intake was examined, and the predictability was good, laying the foundations for the development of biomarker panels.

## 1. Introduction

Identification of new biomarkers of food intake is of high interest and could potentially provide important new tools for dietary compliance monitoring and dietary intake assessment in nutrition and health sciences [1]. These biomarkers have the potential to address limitations associated with the assessment of dietary intake. Food intake biomarkers are single metabolites or a combination of them, reflecting the consumption of either a specific food or food group, displaying a clear time– and dose–response after food intake [2]. The concept of multibiomarker panels has recently emerged as a means of assessing food intake [3,4,5].

Validation of food intake biomarkers is critical for the advancement of their use; a set of eight criteria emerged as a tool to guide validation [1]. These criteria include biological plausibility, dose–response, time–response, robustness, reliability, stability, analytical performance, and inter-laboratory reproducibility [1]. Plausibility reflects a meaningful relationship between the biomarker and food intake; the dose–response aspect establishes whether an increased portion of food results in increased biomarker levels, and the time–response criterion evaluates the kinetic response to intake. With respect to robustness the key question to address is whether the biomarker is still valid following intake of complex meals whereas reliability indicates that there is agreement with self-reported measures of intake. The performance of the analytical method is also a key aspect of validation. Although very few biomarkers of food intake are sufficiently validated, some robust biomarkers of food intake have been identified. Examples include alkylresorcinols, which are biomarkers for wholegrain wheat and rye intakes, and urinary proline betaine, a well-studied biomarker of citrus fruit intake [6,7].

Berries are a group of fruits consumed worldwide and are a good source of polyphenols, micronutrients, and fiber [8]. A recent systematic review examined biomarkers of berry intake and concluded that there is a paucity of validated biomarkers [9]. In discovery studies, the key candidate biomarkers of strawberry intake are pelargonidin conjugates, urolithins, and furaneol and its metabolites [9]. Pelargonidin conjugates (i.e., pelargonidin-3-glucoside, pelargonidin glucuronide, and pelargonidin sulfate) are metabolic products of anthocyanins. They widely exist in pomegranates, plums, and berries and have low abundance in urine [10,11]. Urolithins are included as potential candidates, but their concentrations in urine vary hugely among individuals [12,13,14]. Furaneol and its metabolites are the main strawberry aroma metabolites with high abundances [15]. However, furaneol has been found in kiwi, tomato, and coffee [16]. Mesifurane is an enzymatic methylation product of furaneol, which can be found in mangoes [17,18]. While the above markers are related to strawberry intake, they lack specificity. In most biomarker studies of blueberry intake, anthocyanins, including pelargonidin glucuronide, delphinidin glucuronide, malvidin glucoside, and petunidin glucoside, and phenolic metabolites were identified as putative biomarkers [19,20,21,22]. Furthermore, untargeted metabolomic analysis revealed conjugates of cyanidin, delphinidin, malvidin, benzoic, ferulic, phenylpropionic acid derivatives, and phenyl-valerolactone derivatives as the discriminant metabolites of blueberry intake [23,24,25]. However, similar to the candidate strawberry biomarkers, these compounds are not specific to blueberry consumption. While the above biomarkers may lack specificity, there is the potential for use in a wider biomarker panel for berry consumption. Nonetheless, more specific biomarkers of berry intake are warranted. The objective of this study was to identify biomarkers of mixed strawberry and blueberry intake by combining data from an acute discovery study and a dose–response study.

## 2. Materials and Methods

### 2.1. US–Ireland Discovery Study: Participant Recruitment and Study Design

This discovery study was designed to identify food intake biomarkers of 3 food groups: mixed berries (strawberries and blueberries), green beans, and mixed spinach and lettuce. Ethical approval was obtained from the Human Research Ethics Committee at University College Dublin (UCD) (LS-19-57-Brennan). For the present research, the focus is on the identification of mixed strawberry and blueberry intake biomarkers; therefore, only data related to mixed strawberries and blueberries are reported. The inclusion criteria were healthy men and women aged between 18 and 50 years, a body mass index (BMI) between 18.5 and 30 kg/m^2^, non-smokers, not pregnant or lactating, not taking any medication (except oral contraceptive pill), and not having any diagnosed medical conditions. Interested individuals were screened for eligibility, and those who fulfilled the criteria were asked to sign informed consent before commencing the study. All eligible participants were randomized to consume the 3 test foods in random order, with a one-week break between each visit. The recruitment and screening numbers are presented in Figure S1.

Participants were asked to avoid the consumption of alcohol, medications, and any foods related to the test foods for 24 h prior to the study visit. Following an overnight fast (12 h), participants collected a fasting first void urine sample at home and transported it to the test center in a cool bag on ice. A large portion of the test foods was consumed in the morning with 100 mL of water. The portion of mixed strawberries and blueberries consumed was 192 g of strawberries with 150 g of blueberries. Urine samples were collected at 2, 4, and 6 h following consumption of the foods. Participants did not eat during this 6 h period, but 100 mL of water was provided every 2 h. Following the 6 h urine collection, participants resumed their normal diet but continued to avoid alcohol, medication, and similar foods. A fasting sample the following morning was collected (24 h).

Urine samples were centrifuged at 1800× *g* for 10 min at 4 °C, and 1 mL aliquots were stored at −80 °C until further analysis.

### 2.2. US–Ireland Dose–Response Study

Ethical approval for the dose–response study was granted by the Human Research Ethics Committee at University College Dublin (UCD) (LS-20-24-Brennan). The recruitment strategy and inclusion criteria were the same as the discovery study. Participants were randomized to either consume green beans (n = 16) or mixed strawberries and blueberries and spinach (n = 17). Each group consumed a low, medium, and high portion of the test food over 3 weeks. The 3 portion sizes of mixed strawberries and blueberries were 78 g, 278 g, and 428 g (equal parts strawberries and blueberries). For each portion, participants consumed the food with their evening meal for 4 consecutive days and provided a fasting first-void urine sample on day 5. All test foods were provided by the researchers and participants weighed the exact portions of the test food consumed each evening. Volunteers sent pictures of their plates before and after consuming their evening meal with the test food for compliance. During the 4-day testing period, participants were asked to avoid consuming any foods related to their test food. A 24 h recall was performed with the participants on day 5 to assess compliance with food avoidance. For 24 h prior to sample collection on day 5, participants were also asked to avoid consuming alcohol and medications. Urine samples were processed and stored under the same conditions as the discovery study.

### 2.3. Sample Profiling by LC-MS

The pre-treatment and metabolomic analysis of the urine samples was performed as previously described [26]. The samples were thawed on a roller and centrifuged at 1800× *g* for 5 min at 4 °C. Then, 100 μL samples were added with 100 μL internal standards (malic acid d_3_, methionine d_3_, mysristic acid ^13^C, adipic acid d_4_, and succinic acid d_4_ 10 µg/mL in 20% *v/v* EtOH/Millipore H_2_O), which are isotopes of compounds present in human urine and used to check the reproducibility across batches. Finally, the supernatants were obtained for profiling by LC-MS after vortex and centrifuging. The samples were analyzed by an Agilent LC-QTOF-MS, consisting of a 1290 Infinity II LC system and an Agilent Jetstream (AJS) electrospray ionization (ESI) source coupled to a 6545 QTOF mass spectrometer with the selection of positive and negative ionization modes. A Zorbax eclipse plus C18 2.1 × 5 mm, 1.8 µm column was used as a guard column, and a Zorbax eclipse plus C18 (2.1 × 50 mm, 1.8 µm) column was used for separation. The mobile phase was 0.1% formic acid in water (Eluent A) and 0.1% formic acid in acetonitrile/water (80:20) (Eluent B). Gradient conditions were 1% B (0–1.5 min), 11% B (1.5–9 min), 25% B (9–15 min), 50% B (15–18 min), 99% B (18–18.05 min), 99% B (18.05–21 min), 1% B (21–21.05 min), and 1% B (21.05–23 min). The osmolality of samples was determined by Advanced Instruments Micro Osmometer model 3300 using freezing point depression. Profiled urinary metabolite levels from the LC-MS were normalized to osmolality.

### 2.4. Data Processing and Statistical Analysis

Data were processed using MassHunter Qualitative Analysis (B.07.00 Sp2 Agilent Technologies) software. Multivariate statistical analysis of the LC-MS data was performed using SIMCA 13 (SIMCA Version 13.0.3.0 Umetrics, AB). A partial least squares discriminant analysis (PLS-DA) model examined the differences between 0 h and 4 h post-consumption in both positive and negative modes. These models were used to generate variable importance with projection (VIP) scores, and the top 100 features with a VIP > 1.2 in the positive mode and >1.4 in the negative mode were considered of interest.

Furthermore, some potential metabolite biomarkers were selected from current research papers and reviews pertaining to mixed strawberries and blueberries. The intensity of the biomarkers exhibiting a clear time course was analyzed using repeated measures ANOVA (*p* < 0.05) to demonstrate a significant increase associated with the consumption of mixed strawberries and blueberries. Additionally, an internal molecular network was established using the Global Natural Products Social Molecular Networking (GNPS) platform using data from pooled urine samples from the discovery study [27]. The spec m/z values of the nodes represent the metabolites with similar m/z values after intake of mixed strawberries and blueberries. Potential biomarkers from previous literature and those biomarkers showing significant differences between fasting and 4 h postprandial in the discovery study, were considered of interest. Given the internal network where these nodes are located, it is hypothesized that an increase in the intensity of the nodes of interest would also affect the adjacent nodes. Therefore, the three nodes adjacent to them were included to observe the intensity changes following additional berry consumption. Their time– and dose–response patterns were evaluated through a repeated measures ANOVA (*p* < 0.05).

The daughter MS/MS fragments with different collision energies (10 eV, 20 eV, and 40 eV) were generated from the parent compounds, and fragmentation matching to the candidate spectrum in the METLIN, HMDB, GNPS, MassBank Europe, and MassBank of North America (MoNA) databases was performed. Metabolite identifications were assigned at four levels with accordance to metabolomics standards initiative (MSI): Level I (identified compounds); Level II (putatively annotated compounds); Level III (putatively characterized compound classes); Level IV (unknowns) [28].

### 2.5. Multiple Biomarkers for Prediction of Intake

To examine the ability of the panel of biomarkers to predict intake, the multiMarker application was used [29]. A test set consisting of 2% of the data from the dose–response study was used to examine the ability of the biomarkers to predict the intake of mixed strawberries and blueberries. A training set of 98% of the data was used to establish the model. This procedure was repeated ten times for each intake portion.

## 3. Results

### 3.1. Identification of Features Associated with Mixed Strawberry and Blueberry Intake

In total, 27 participants were recruited, and 25 participants consumed mixed strawberries and blueberries in the discovery study. They included 13 males and 12 females, aged 18–49 years, with mean BMI of 23.46 kg/m^2^. In the dose–response study, 7 males and 10 females aged 20–50 years, with mean BMI of 22.02 kg/m^2^ completed the study. The participant details are reported in Table S1.

**Figure 1:**
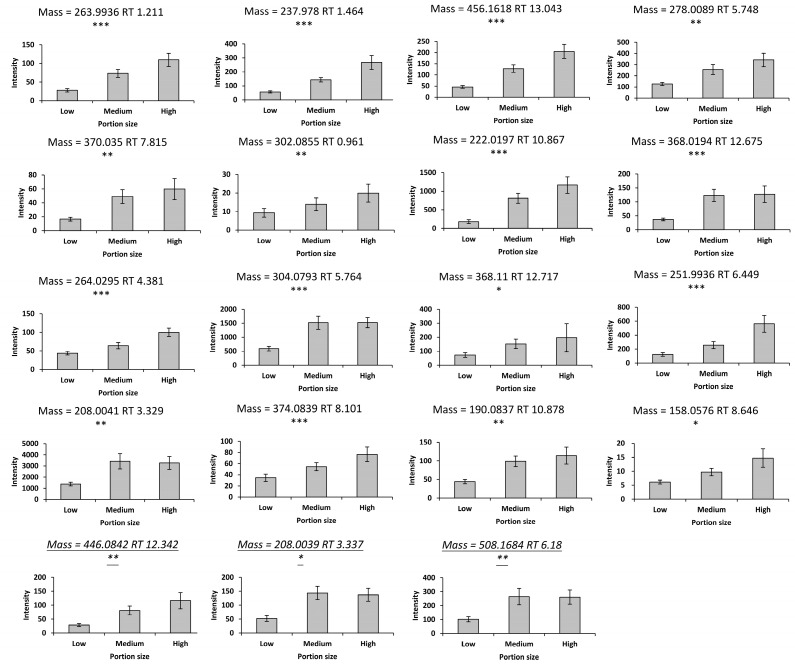
Metabolites with significant dose–response relationships (* *p* < 0.05, ** *p* < 0.01, and *** *p* < 0.001). The feature with a mass = 368.11 is significant between the low and medium portions. The metabolites in the bottom row were acquired in the positive mode. Values are the means ± SEM. *X*-axis values represent different portions of berry intake; low, medium, and high portions of mixed strawberries and blueberries were 78 g, 278 g, and 428 g (equal parts strawberries and blueberries). *Y*-axis values represent the peak height normalized by osmolality. Significance was assessed using repeated measures ANOVA.

A total of 913 features in the negative mode and 719 features in the positive mode were obtained from the urine samples in the discovery study. Principal component analysis of the urine LC-MS data revealed separation of the samples according to time following consumption of mixed strawberries and blueberries. The PLS-DA models were built using the data from fasting and 4 h postprandial samples with *R^2^X* = 0.281, *Q^2^* = 0.769 in the negative mode, and *R^2^X* = 0.399, *Q^2^* = 0.663 in the positive mode (Figure S2). Potential interesting features were identified using the VIP scores from both models. In total, 39 features in the negative mode and 15 features in the positive mode showed increased responses following consumption of mixed strawberries and blueberries (Figures S3 and S4). Of these, there were 15 features in the negative mode and 3 features in the positive mode with significant dose–response relationships (*p* < 0.05) over the consumption of different portion sizes of mixed strawberries and blueberries (see Figure 1). Furthermore, one feature showed significance between low and medium portions.

**Figure 2:**
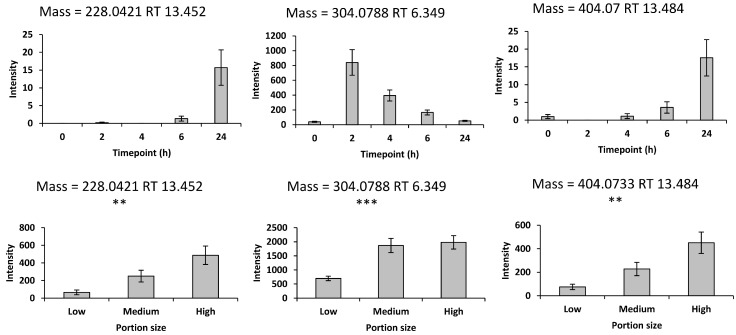
The supplementary biomarkers from previous research with time– and dose–response relationships in the positive mode. Values are the means ± SEM. The top panel represents the time-course plots with the *X*-axis indicating the timepoints after intake of 192 g of strawberries with 150 g of blueberries; *Y*-axis values represent the peak height. The bottom panel represent the dose–response data with the *X*-axis values representing different portions of mixed strawberries and blueberries intake, specifically 78 g (low), 278 g (medium), and 428 g (high), consisting of equal parts strawberries and blueberries. *Y*-axis values represent the peak height normalized by osmolality. Repeated measures ANOVA was conducted to assess significant changes in the intensities of the biomarkers after consuming the 3 different portions of mixed strawberries and blueberries ** *p* < 0.01, and *** *p* < 0.001).

Previous literature identified furaneol, pelargonidin and urolithin and their glucuronides and sulphates as potential biomarkers of mixed strawberry and blueberry intake in urine sample. Viewing the time– and dose–response of these potential biomarkers, 3 of them in the positive mode were added to the biomarker panel (Figure 2). Molecular networking builds on the fundamental observation by GNPS that two structurally related molecules share fragment ion patterns when subjected to MSMS fragmentation methods to provide a chemical insight for biological questions [30]. Herein, an internal network of berries was created by connecting related mass spectra from a pooled urine sample, which consisted of equal volumes of each sample in the dose–response study, by GNPS. After monitoring the nodes of interest within the three nodes adjacent to the 57 nodes representing the 57 potential biomarkers (54 features from the discovery study and three features from the literature review), only one feature displayed a significant difference in response to different consumption of mixed strawberries and blueberries. Following this an additional biomarker (Figure S5) was added to the biomarker panel in network 30 (Figure S6). Overall, the final panel comprised 17 biomarkers in the negative mode and 6 biomarkers in the positive mode (Table 1).

### 3.2. Identification of Biomarkers of Mixed Strawberry and Blueberry Intake

The identification of M04 was based on the authentic standard syringic acid in the negative mode (Figure S7). The difference in the mass between syringic acid and M04 syringic acid sulfate was ≈79.96 Da, which corresponds to the mass of a sulfate group. The feature M19 was identified as urolithin A using an authentic standard. The feature M21 with a difference in mass of ≈176.03 Da between its parent ion and M19, was identified as urolithin A glucuronide (Figure S8). The major fragments of M16 matched well with those of the authentic standard pelargonidin chloride (Figure S9). After fragmentation with a collision energy of 20 eV, M17 lost a neutral fragment of ≈79.96 Da and M20 showed an obvious difference in mass between the core structure and the precursor of ≈176.03 Da. The core structures left on M17 and M20 matched well with the authentic standard furaneol in the positive mode (Figure S10). Correspondingly, the core structures of two sulfated and glucuronidated biomarkers in the negative mode also matched to furaneol (Figure S11). Hence, the total number of unique biomarkers was 21.

M01, M03, M06, M07, M09, and M10 were putatively identified according to previous literature. Backups for these assignments were from MS/MS fragmentation matches in databases. For the M02 without the sulphate, there was a good match with the predicted LC-MS/MS spectrum for zymonic acid in HMDB, with scores of 0.96 (10 eV) and 0.72 (25 eV). M05 was putatively identified as folerogenin (Metlin ID: 92381), using Metlin (score: 0.5841 using 40 eV collision energy). For M08, the metabolite was putatively predicted as 3-methoxy-4-hydroxyphenylglycol sulfate by HMDB (score: 0.91 using 25 eV collision energy) with confirmation of the presence of a sulfate group in the fragmentation pattern. M11 can be separated into two moieties, where one is sulfate (≈79.96 Da), and the m/z of the other one is 171.0292 in the negative mode, which is the exact m/z of 3-dehydroshikimate provided by MoNA [31,32]. M13 was assigned as 3-hydroxysuberic acid by HMDB, and matched the product ions previously reported [33]. M14 was putatively identified as isopropylmaleic acid by spectrum matching in HMDB with scores of 0.93 (10 eV), 0.90 (20 eV), and 0.99 (40 eV). M12 was putatively identified as an unknown moiety with the formula of C_9_H_10_O_5_ attached to a glucuronide because of a neutral loss at ≈176.03 Da. Because of the lack of similar fragmentation characteristics in databases, M15 remained unknown. M18 was putatively identified as tryptophan furaneol glucuronide, because the mass of M18 was the exact mass of two moieties. One is furaneol glucuronide with similar fragmentation; the other is L-tryptophan.

### 3.3. Prediction of Intake Using a Biomarker Panel

A panel of seven biomarkers was created by selecting the biomarkers that were significantly different among each of the three portions. The details of these seven biomarkers are shown in Table S2. Using the training set, the estimated model from the multiMarker framework was used to predict berry intake for the test data [34]. Comparison between the predicted and intake portions revealed good agreement between the two (Table 2). For the low and medium portions, excellent agreements were observed. However, there was some disagreement for the large portion of berries. It should be noted that while participants were provided with the berries to eat, we cannot guarantee that the full portions were consumed.

## 4. Discussion

There is increased interest in biomarkers of food intake, with the potential of these biomarkers to improve assessments of dietary intake. Here, we report a panel of biomarkers identified from a discovery study and validated in a dose response study. Importantly, the multibiomarker panel predicted intake with good agreement, paving the way forward for its use in the assessment of food intake.

Emerging literature suggests that it is difficult to quantify food intake using a single biomarker, owing to the overlapping range of compounds in foods. Thus, multibiomarker panels generated with two or more biomarkers together can add sensitivity and specificity to the intake prediction [34]. Herein, a metabolomics-based approach generated a biomarker panel of 21 urinary metabolites which together represented a diverse range of chemical structures. Using the multiMarker framework, the relationship between the panel of biomarkers and mixed strawberry and blueberry intake was modeled. The estimates from this model were then used to predict intake using the biomarker data only. On the whole, good agreement was obtained demonstrating the potential of the biomarker panel. Furthermore, the 95% intervals highlight the uncertainty associated with each prediction and can be valuable when assessing intake.

Biological plausibility is a key aspect in the validation of biomarkers of food intake. With respect to the biomarker panel, there is a strong argument to link the panel with berry intake. The most prevalent secondary metabolites in strawberry fruit are phenolic compounds, which have at least one phenol unit (aromatic organic ring) in their chemical structures. Generally, berries contain high concentrations of phenolic compounds (up to 750 μg/g fresh weight) [35]. The metabolites in the biomarker panel covered phenolic acids (Group 1 and Group 4), flavonoids (Group 3), and furanones (Group 2); of these, phenolic acids and flavonoids belong to phenolic compounds [36]. To reveal the inner relationship of the biomarkers, four metabolic pathways associated with metabolites in the biomarker panel were established and expressed as groups.

**Figure 3:**
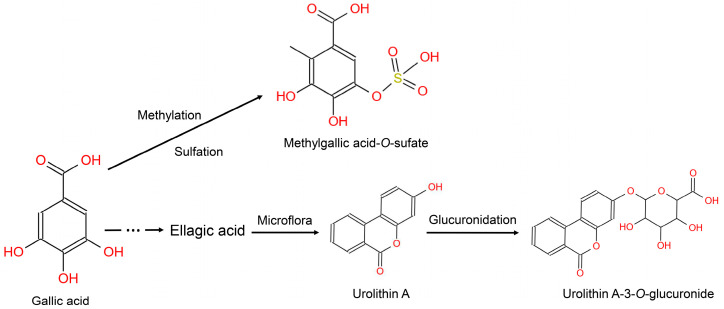
Metabolism of gallic acid in humans.

Phenolic acids widely exist in berry fruits in high contents [37]. There are 85 mg/100 g phenolic acids in blueberry, with syringic acid as the predominant of the insoluble phenolic acids; in addition, there are 10–18 mg/100 g phenolic acids in strawberries, with high concentrations of gallic acid. The metabolism of phenolic acid gives rise to various metabolites—many of which were captured in our biomarker panel, adding to the biological plausibility of the biomarkers (Figure 3, Group 1). Of note is the biomarker syringic acid which can be metabolized to syringic acid sulfate [38]. Gallic acid can be converted into ellagic acid which can be transformed by the gut microbiota to produce urolithins, however, there is large interindividual variability among humans with respect to urolithin production [39]. This was additionally confirmed in our work; indeed, urolithin did not emerge as a biomarker using the untargeted approach—it was added following a review of the literature.

**Figure 4:**
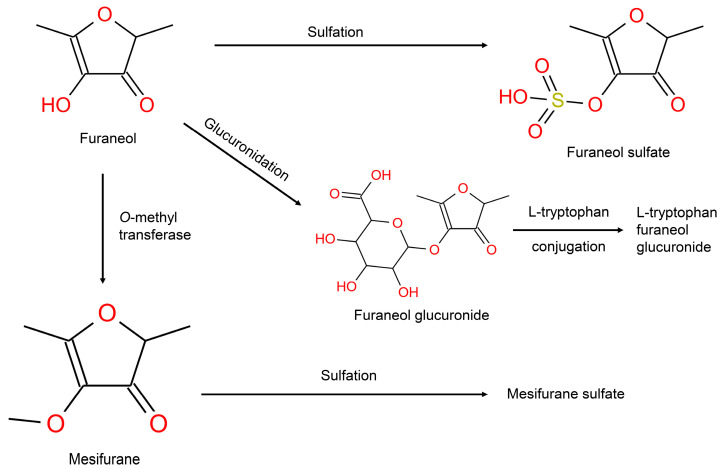
Metabolism of the metabolite furaneol in humans.

Furaneol, is an important volatile in strawberry with a high concentration (up to 55 mg/kg fresh weight) and low odor threshold (10 μg/L water for odor) [40]. The endogenous metabolic pathway of furaneol is shown in Figure 4 (Group 2). The metabolites related to furaneol metabolism were present in high intensities in both the positive and negative modes. Mesifurane is a significant flavorant in arctic brambles, mangoes, strawberries, and many other fruits and berries and is an enzymatic methylation product of furaneol [20]. In the present work, its conjugation with sulfate emerged as a biomarker of mixed strawberry and blueberry intake which agrees with previously reported studies [41,42].

Blueberries contain a significant level of anthocyanins, which can account for up to 60% of the total polyphenolics in ripe blueberries [43,44]. Dihydrokaempferol serves as a precursor for anthocyanidins, anthocyanins, catechins, etc. [42] Leucopelargonidin and pelargonidin are water-soluble anthocyanidins derived from dihydrokaempferol. Dihydrokaempferol sulfate has been mentioned as a urinary biomarker following intake of strawberry [41]. Pelargonidin glucuronide was reported as a biomarker following intake of blueberries [19]. Our research agrees with the literature and adds evidence for the potential of pelargonidin glucuronide as a biomarker of intake. Furthermore, leucopelargonidin sulfate emerged as a biomarker for mixed strawberries and blueberries.

Quinic acid and shikimate are major organic acids in blueberries [45,46]. Downstream metabolites such as 3-caffeoylquinic acid were reported as a potential urinary biomarker of berries [47]. In the present study, feruloylquinic acid, a derivative of quinic acid, also featured as a potential biomarker of intake.

Using biomarkers as objective measures of intake has garnered a lot of attention in recent years. Combining biomarkers into a multimarker panel can help address some of the limitations associated with certain biomarkers such as lack of specificity. Our work provides the initial evidence that multimarker panels can predict berry intake. However, further work is needed to test this in a larger and more diverse group of individuals. Furthermore, combining these biomarkers with self-reported data has the potential to enhance the accuracy of dietary assessments. The present work should form the basis for this in the future.

## 5. Conclusions

Most of the biomarkers of the 21-multimarker panel have high biological plausibility as biomarkers of berry intake. From the panel, a prediction model was established, which performed well in predicting berry intake. The development of methods that quantitively measure these biomarkers and assessment of the within-person variation over time will be important next steps.

## Figures and Tables

**Table 1:** Identification of biomarkers following intake of mixed strawberries and blueberries.

M	RT	Mass	M/Z	Ion	MS/MS	Suggested Metabolite	Formula	Pathway
M01	1.211	263.9936	262.9866	[M − H]^−^	79.9564, 96.9603, 167.0204, 183.0290	Methylgallic acid-*O*-sulfate II	C_8_H_8_O_8_S	Group 1
M02	1.464	237.978	236.9705	[M − H]^−^	41.0031, 55.0194, 157.0132	Zymonic acid sulfate II	C_6_H_6_O_8_S	-
M03	13.043	456.1618	455.1543	[M − H]^−^	113.0245, 175.0231, 217.1220, 279.1213	Hydroxy-abscisic acid glucuronide II	C_21_H_28_O_11_	-
M04	5.748	278.0089	277.0015	[M − H]^−^	182.0209, 197.0447	Syringic acid sulfate I	C_9_H_10_O_8_S	Group 1
M05	0.961	302.0855	301.0781	[M − H]^−^	124.0146, 167.0200	Folerogenin II	C_16_H_14_O_6_	-
M06	10.867	222.0197	221.0117	[M − H]^−^	79.9569, 126.0315, 141.0552	Mesifurane sulfate II	C_7_H_10_O_6_S	Group 2
M07	12.657	368.0194	367.0118	[M − H]^−^	93.0341, 165.0189, 191.0549, 287.0542	Dihydrokaempferol-7-*O*-sulfate II	C_15_H_12_O_9_S	Group 3
M08	4.381	264.0295	263.0219	[M − H]^−^	168.0429, 183.0656	3-Methoxy-4-hydroxyphenylglycol sulfate II	C_9_H_12_O_7_S	-
M09	7.815	370.035	369.0274	[M − H]^−^	96.9596, 153.0186, 289.0700	Leucopelargonidin sulfate II	C_15_H_14_O_9_S	Group 3
M10	12.717	368.11	367.1025	[M − H]^−^	93.0342, 134.0366, 173.0443, 287.0542	Feruloylquinic acid II	C_17_H_20_O_9_	Group 4
M11	6.449	251.9936	250.9859	[M − H]^−^	79.9568, 171.0292	3-Dehydroshikimate sulfate II	C_7_H_8_O_8_S	Group 4
M12	8.101	374.0839	373.0764	[M − H]^−^	113.0245, 197.0447	Unknown glucuronide III	C_15_H_18_O_11_	-
M13	10.878	190.0837	189.0764	[M − H]^−^	107.0495, 129.0552, 149.0601	3-Hydroxysuberic acid II	C_8_H_14_O_5_	-
M14	8.646	158.0576	157.0498	[M − H]^−^	97.0663, 115.0758	Isopropylmaleic acid II	C_7_H_10_O_4_	-
M15	0.699	176.0034	174.9554	[M − H]^−^	44.9981, 86.9764, 130.9660	Unknown IV	-	-
M16	12.342	446.0842	447.0917	[M + H]^+^	271.0607	Pelargonidin glucuronide I	C_21_H_19_O_11_	Group 3
M17	3.337	208.0039	209.0114	[M + H]^+^	43.0173, 57.0327, 129.0549	Furaneol sulfate I	C_6_H_8_O_6_S	Group 2
M18	6.18	508.1684	509.1758	[M + H]^+^	129.0547, 146.0595, 188.0705, 205.0969, 305.0875	L-tryptophan furaneol glucuronide III	C_23_H_28_N_2_O_11_	Group 2
M19	13.452	228.0421	229.049	[M + H]^+^	128.0618, 157.0647, 185.0595	Urolithin A I	C_13_H_8_O_4_	Group 1
M20	6.349	304.0788	305.0862	[M + H]^+^	43.016, 57.0335, 95.0131, 129.0547	Furaneol glucuronide I	C_12_H_16_O_9_	Group 2
M21	13.484	404.0733	405.0808	[M + H]^+^	229.0512	Urolithin A-3-*O*-glucuronide I	C_19_H_16_O_10_	Group 1

Roman numerals indicate the level of identification, where I represents level I identifications, II represents level II identifications, III represents level III identifications, IV represents level IV identification. Four metabolic pathways associated with metabolites in the biomarker panel were established: Group 1 and Group 4 represent the metabolism of phenolic acids; Group 2 and Group 3 are involved in the metabolism of furanones and flavonoids, respectively. M: metabolite, RT: retention time, M/Z: mass to charge ratio.

**Table 2:** Comparison between the mixed strawberry and blueberry intake and the predicted mixed intake.

Observation	Intake (g)	Predicted Intake (g)	Standard Deviation	2.5% Percentile	97.5% Percentile
1	**78**	**79.1**	8.9	64.0	99.9
2	**78**	**77.6**	8.6	59.2	94.2
3	**78**	**77.0**	8.6	57.2	92.1
4	**78**	**77.7**	8.3	60.7	94.2
5	**78**	**78.2**	8.5	61.5	95.7
6	**78**	**77.2**	8.7	58.2	92.4
7	**78**	**78.7**	8.7	62.9	98.0
8	**78**	**77.5**	8.5	59.0	93.1
9	**78**	**77.5**	8.7	59.3	93.9
10	**78**	**77.6**	8.7	59.0	94.3
11	278	78.5	8.7	61.9	97.1
12	**278**	**277.5**	5.6	265.4	288.0
13	**278**	**278.3**	5.6	267.1	289.6
14	**278**	**276.6**	6.3	262.3	286.6
15	**278**	**277.1**	5.7	264.5	287.2
16	**278**	**276.6**	6.3	262.2	286.6
17	**278**	**278.4**	5.8	267.1	290.4
18	**278**	**278.2**	5.7	266.9	289.6
19	**278**	**278.7**	5.7	268.0	291.0
20	278	77.8	8.6	59.7	94.1
21	428	277.5	5.6	265.4	288.3
22	428	80.1	11.6	66.5	111.1
23	428	278.7	5.8	268.2	291.1
24	**428**	**427.2**	6.0	414.6	437.7
25	**428**	**427.3**	5.6	415.4	437.6
26	**428**	**429.3**	6.3	419.0	443.6
27	**428**	**430.0**	9.02	420.0	447.3
28	428	278.1	5.64	267.3	289.6
29	**428**	**426.7**	6.15	413.2	436.8
30	**428**	**430.8**	27.1	421.0	507.0

The agreements are highlighted in bold. Predicted intakes are estimated with posterior median and 95% credible interval.

## Data Availability

The raw data supporting the conclusions of this article will be available from the corresponding author upon reasonable request.

## Supplementary Materials

The following supporting information can be downloaded at: https://www.mdpi.com/article/10.3390/metabo14090505/s1, Figure S1: Flow diagram of recruitments for the discovery study; Figure S2: PLS-DA of LC-MS/MS urine data of time point 0 h in comparison with 4 h post-consumption for mixed berries; Figure S3: Plots of urinary LC-MS spectral intensities of 39 features in the negative mode that increased over time following consumption of mixed berries; Figure S4: Plots of features showing increasing response to time following consumptions of mixed berries; Figure S5: The supplementary biomarkers from networks established by GNPS with time– and dose–response relationships in the negative mode; Figure S6: The details on the confirmation of the feature in network 30 by a comparison of the fragments in a pooled urine sample after mixed berry intake and fragments in GNPS; Figure S7: Identification of M04 as syringic acid sulfate by the authentic standard of syringic acid in the negative mode; Figure S8: Identification of M19 as urolithin A and M21 as urolithin A-3-*O*-glucuronide by the authentic standard of urolithin A in the positive mode; Figure S9: Identification of M16 as pelargonidin glucuronide by the authentic standard of pelargonidin chloride in the positive mode; Figure S10: Identification of M17 and M20 as furaneol sulfate and furaneol glucuronide by the authentic standard furaneol in the positive mode; Figure S11: Identification of biomarkers as furaneol sulfate and furaneol glucuronide by authentic standard furaneol in the negative mode; Table S1: Subject characteristics; Table S2: Seven biomarkers with significant differences among each of the three portions.
