# Supplementary material for: Urinary Biomarkers of Strawberry and Blueberry Intake
Source: Metabolites. 2024 Sep 18;14(9):505. doi: 10.3390/metabo14090505 (PMC11434597; doi:10.3390/metabo14090505)
Supplement: Supplementary file 1 [file metabolites-14-00505-s001.zip › metabolites-3172018-supplementary.pdf]

Supplementary material

Urinary biomarkers of berry intake

Ya Gao[1,2], Rebecca Finlay[1,2], Xiaofei Yin[1,2], Lorraine Brennan[1,2]*

1. UCD School of Agriculture and Food Science, Institute of Food and Health, University College Dublin, Belfield, Dublin 4, Ireland

2. UCD Conway Institute, University College Dublin, Belfield, Dublin 4, Ireland

E-mail: lorraine.brennan@ucd.ie

Fax: +353 1 716 6815

Keywords: biomarkers, food intake, metabolomics, mixed berries

Table S1. Subject Characteristics

Characteristics	Discovery study (*N* = 25)	Dose-response study (*N* = 17)
Gender	13 M 12 F	7 M 10 F
Age (years)	23.8 ± 6.89	28.8 ± 9.38
BMI (kg/m^2)	23.5 ± 2.67	22.0 ± 2.23
W:H	0.81 ± 0.06	0.76 ± 0.06

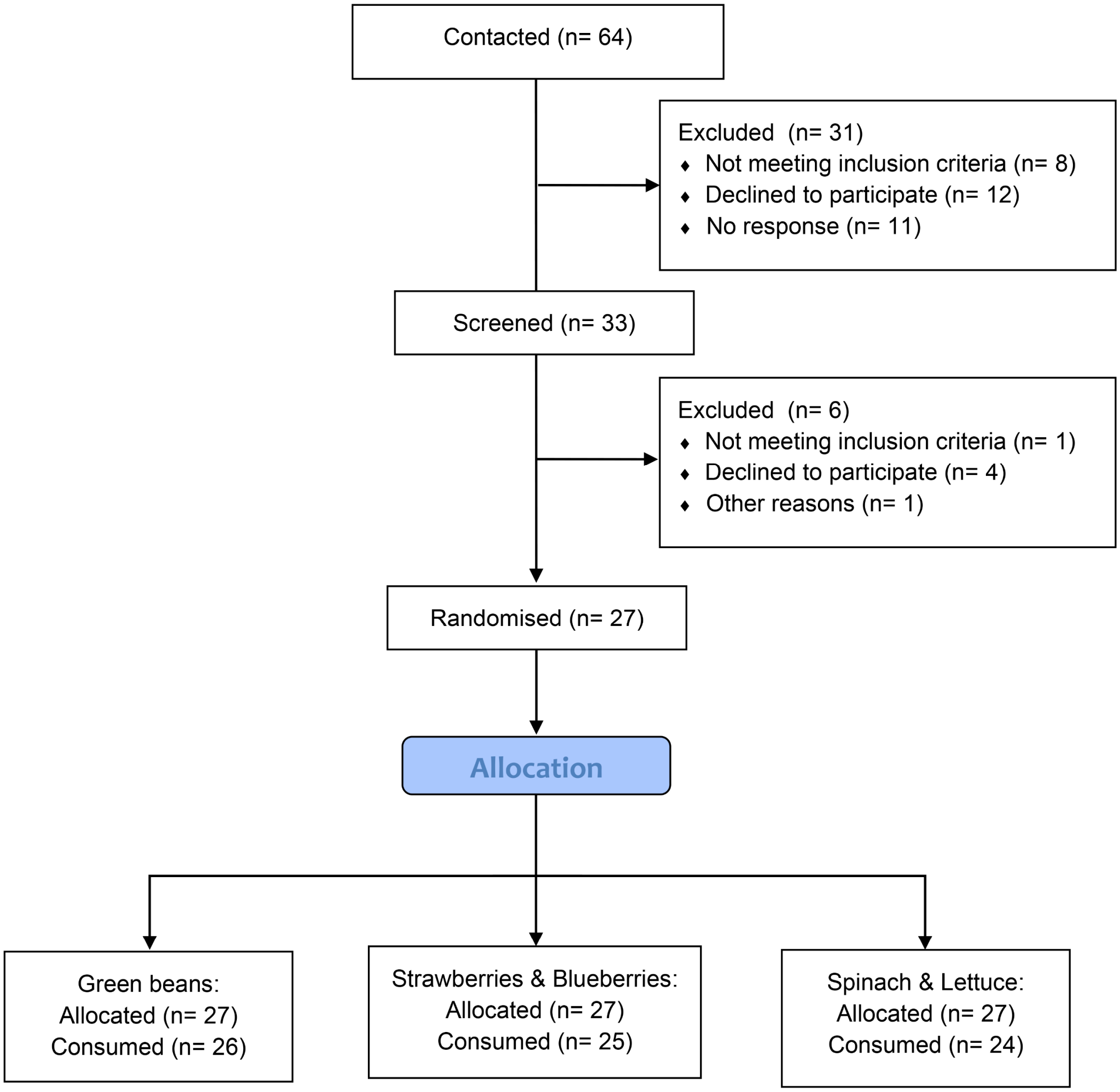

Figure S1. Flow diagram of recruitments for the discovery study.

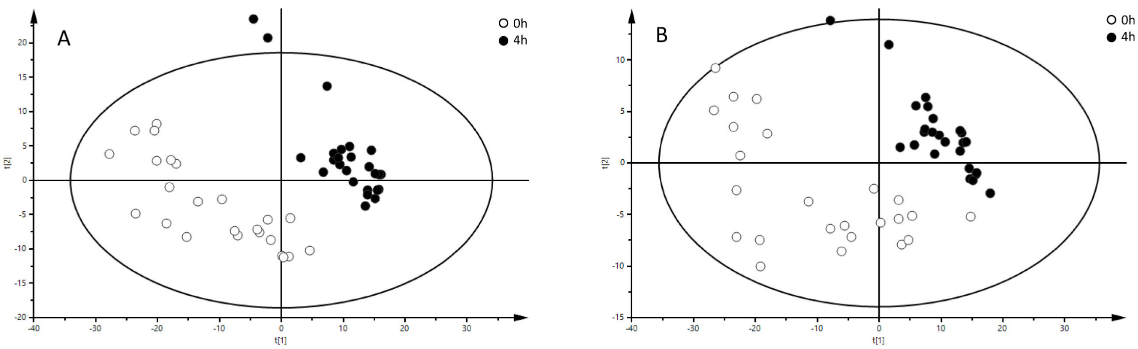

Figure S2. PLS-DA of LC-MS/MS urine data of time point 0 h in comparison with 4 h post-consumption for mixed berry in negative mode (A) with R^2X at 0.281, and Q^2 at 0.769 and in positive mode (B) with R^2X at 0.399, and Q^2 at 0.663.

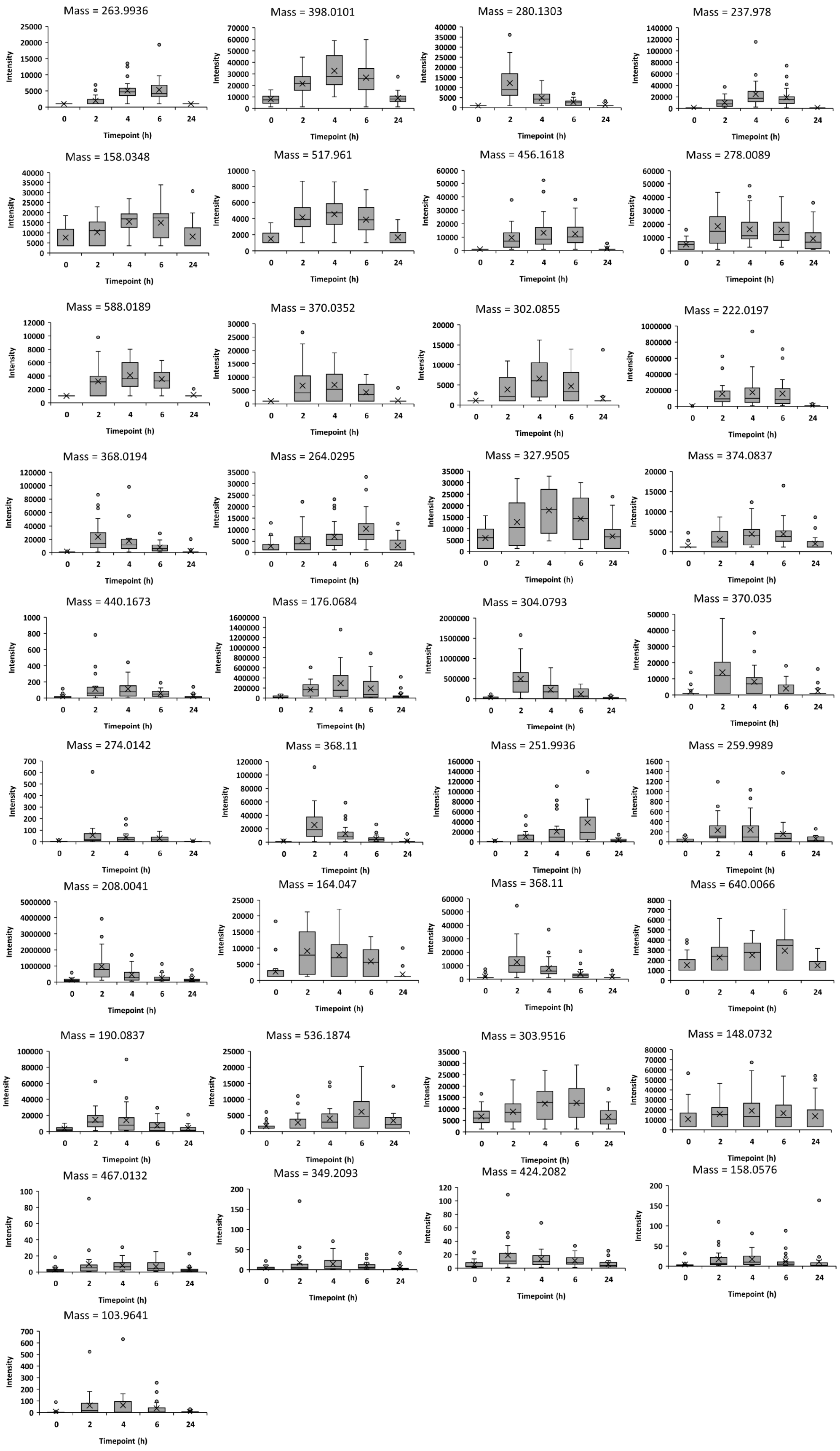
Mass = 263.9936
Mass = 398.0101
Mass = 280.1303
Mass = 237.978
Mass = 158.0348
Mass = 517.961
Mass = 456.1618
Mass = 278.0089
Mass = 588.0189
Mass = 370.0352
Mass = 302.0855
Mass = 222.0197
Mass = 368.0194
Mass = 264.0295
Mass = 327.9505
Mass = 374.0837
Mass = 440.1673
Mass = 176.0684
Mass = 304.0793
Mass = 370.035
Mass = 274.0142
Mass = 368.11
Mass = 251.9936
Mass = 259.9989
Mass = 208.0041
Mass = 164.047
Mass = 368.11
Mass = 640.0066
Mass = 190.0837
Mass = 536.1874
Mass = 303.9516
Mass = 148.0732
Mass = 467.0132
Mass = 349.2093
Mass = 424.2082
Mass = 158.0576
Mass = 103.9641
Intensity
Timepoint (h)

Figure S3. Plots of urinary LC-MS spectral intensities of 39 features in negative mode that increased over time following consumption of mixed berries. *X*-axis values represent the timepoints (0, 2, 4, 6, 24 h) after intake of 192 g of strawberries with 150 g of blueberries; *Y*-axis values represent peak height.

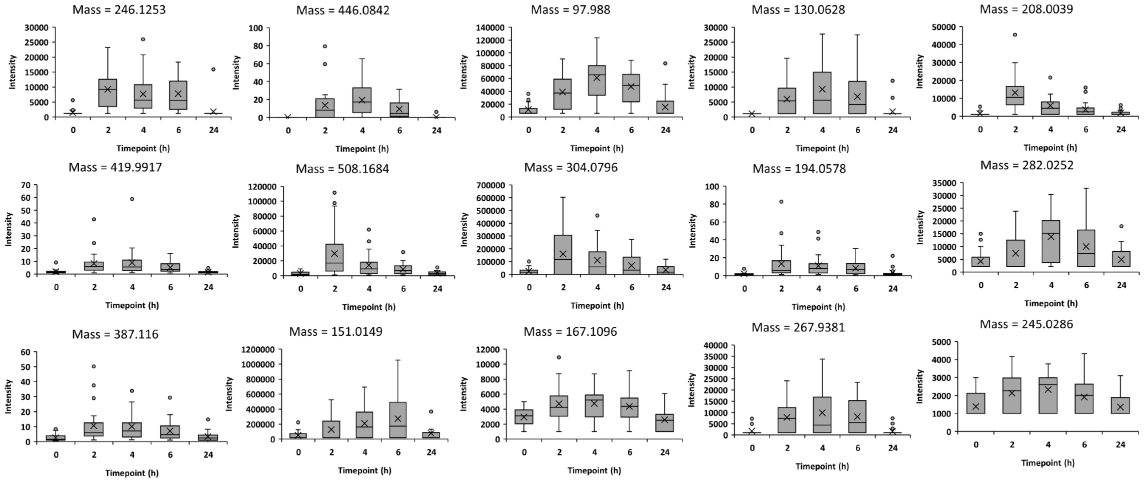

Figure S4. <u>Plots of features showing increasing response to time following consumptions of mixed berries</u>~~Plots of urinary LC-MS spectral intensities of 15 features in positive mode that increased over time following consumption of mixed berries~~. X-axis values represent the timepoints (0, 2, 4, 6, 24 h) after intake of 192 g of strawberries with 150 g of blueberries; Y-axis values represent peak height.

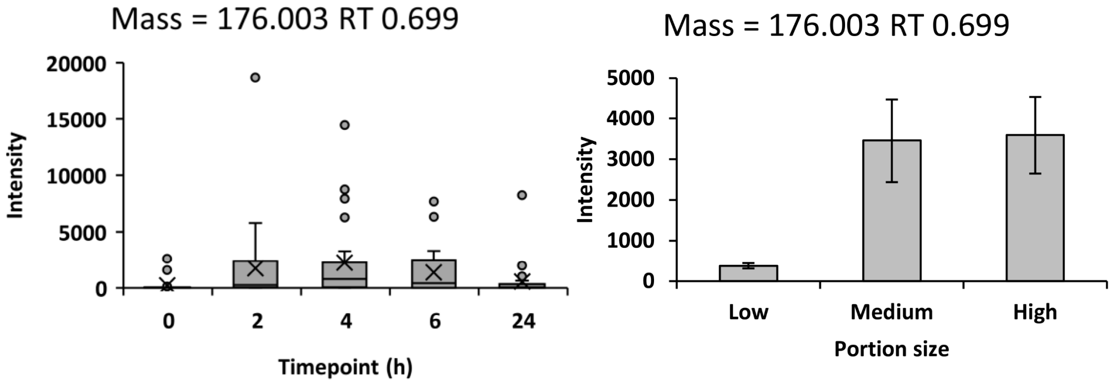

Figure S5. The supplementary biomarkers from networks established by GNPS with time– and dose–response relationships in the negative modeThe supplementary biomarkers from networks established by GNPS with time- and dose- response relationships in negative mode. Values are mean ± SEM. In the figures of timeline plot, X-axis values represent the timepoint after intake 192 g of strawberries with 150 g blueberries; Y-axis values represent peak height. In the figures of dose-response plot, *X*-axis values represent different portion of intake; Low, medium, and high portion of mixed berries were 78 g, 278 g, and 428 g (equal parts strawberries and blueberries). *Y*-axis values represent peak height normalized by osmolality.

Network 30

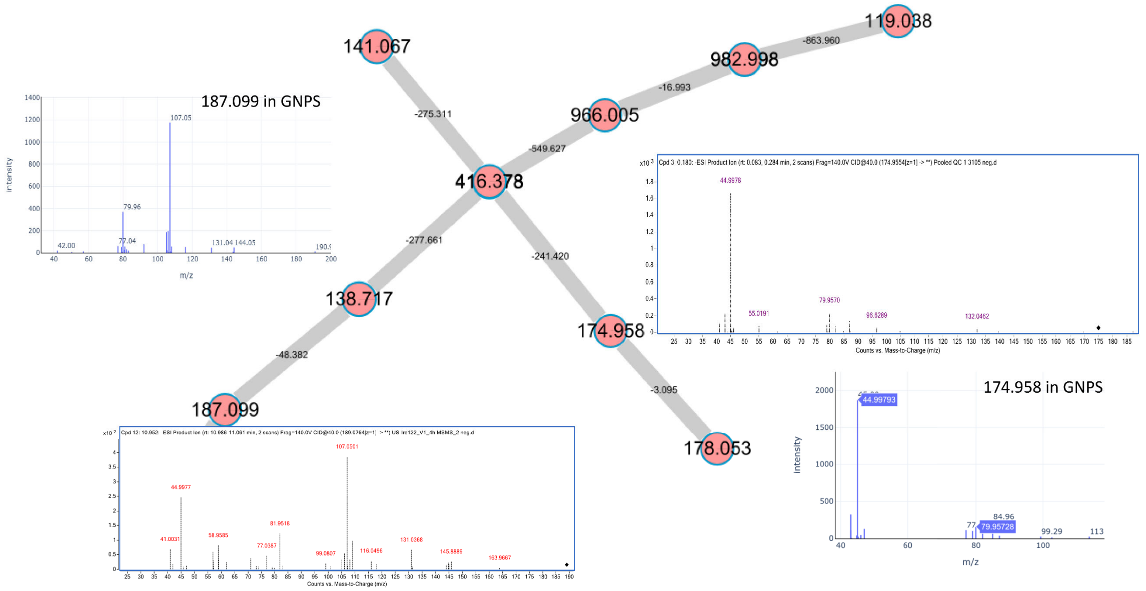

Figure S6. The details on the confirmation of the feature in network 30 by a comparison of the fragments in a pooled urine sample after mixed berry intake and fragments in GNPS~~The screenshot of Network 30 from the molecular networking of a pooled urine sample after mixed berries intake established by GNPS. The interesting node and the adjacent nodes are confirmed by fragmentation matches.~~

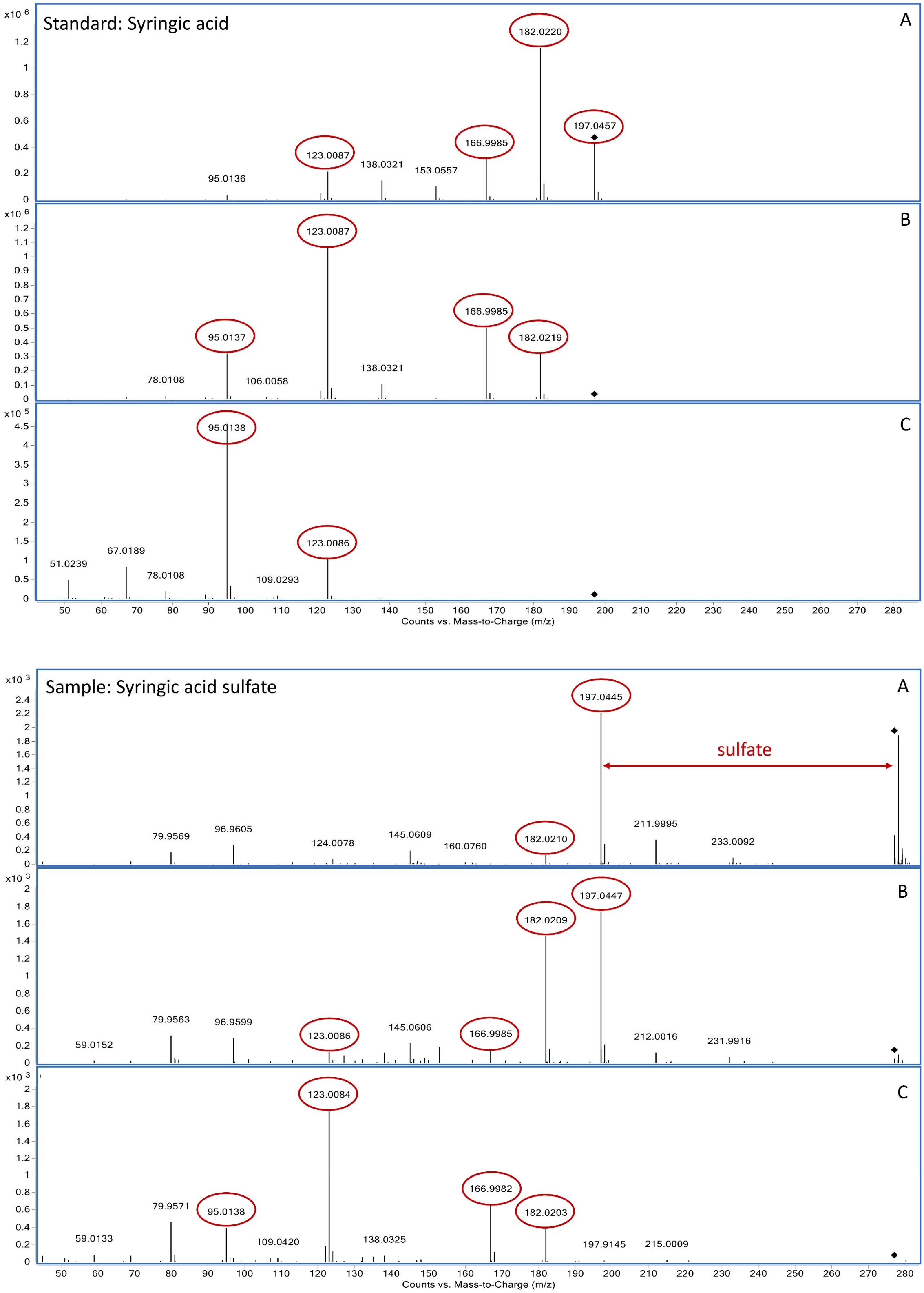

Figure S7. Identification of M04 as syringic acid sulfate by the authentic standard of syringic acid in negative mode. A, B, and C represent the collision energy of 10, 20, and 40 eV respectively on

selected precursors. The red circles indicate identical feature fragments in both the urine sample and the standard.

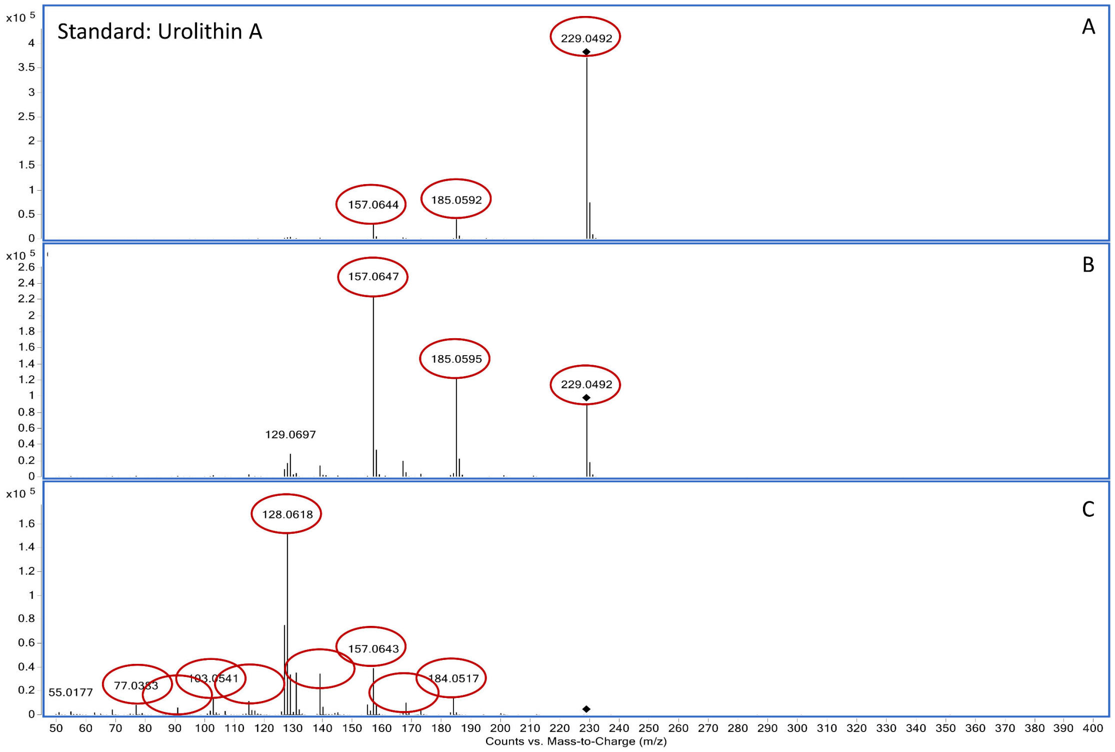
Standard: Urolithin A
A
229.0492
157.0644
185.0592
x10 5
B
157.0647
185.0595
229.0492
129.0697
x10 5
C
128.0618
157.0643
184.0517
77.0333
103.0541
55.0177
x10 5
Counts vs. Mass-to-Charge (m/z)

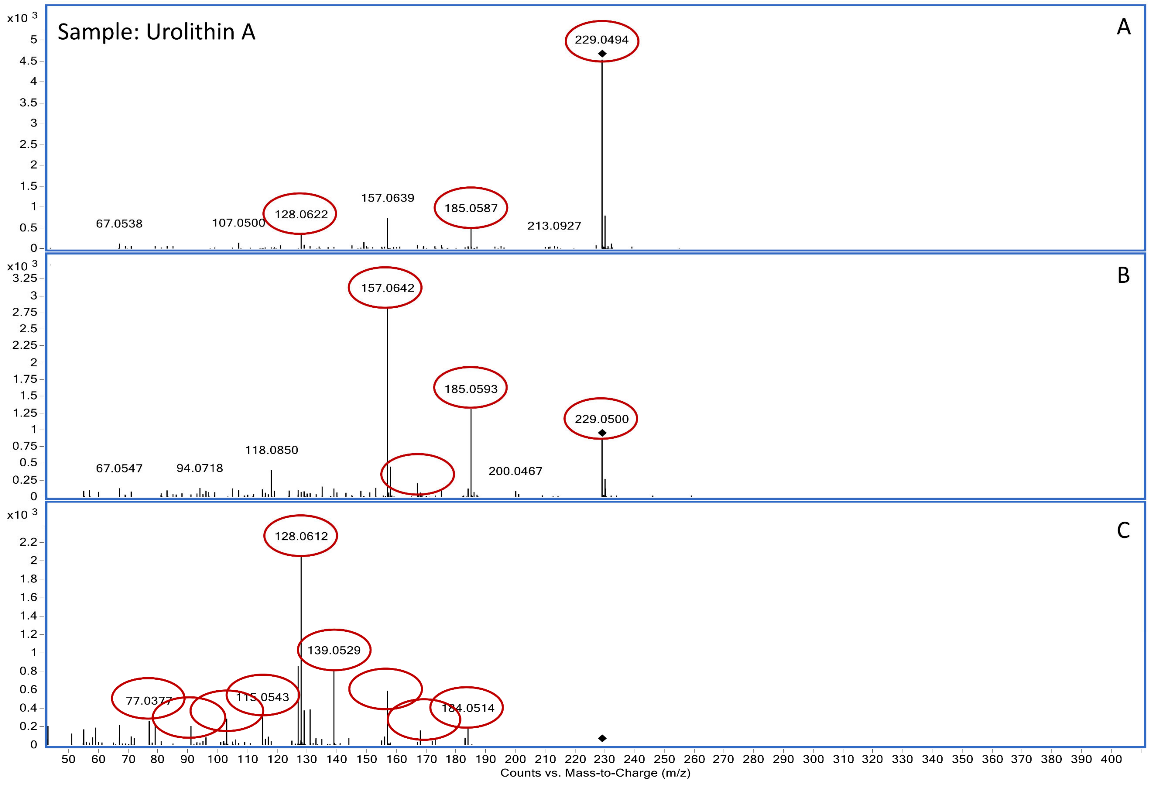
Sample: Urolithin A
A
229.0494
67.0538
107.0500
128.0622
157.0639
185.0587
213.0927
x10 3
B
157.0642
185.0593
229.0500
67.0547
94.0718
118.0850
200.0467
x10 3
C
128.0612
139.0529
77.0377
115.0543
184.0514
x10 3
Counts vs. Mass-to-Charge (m/z)

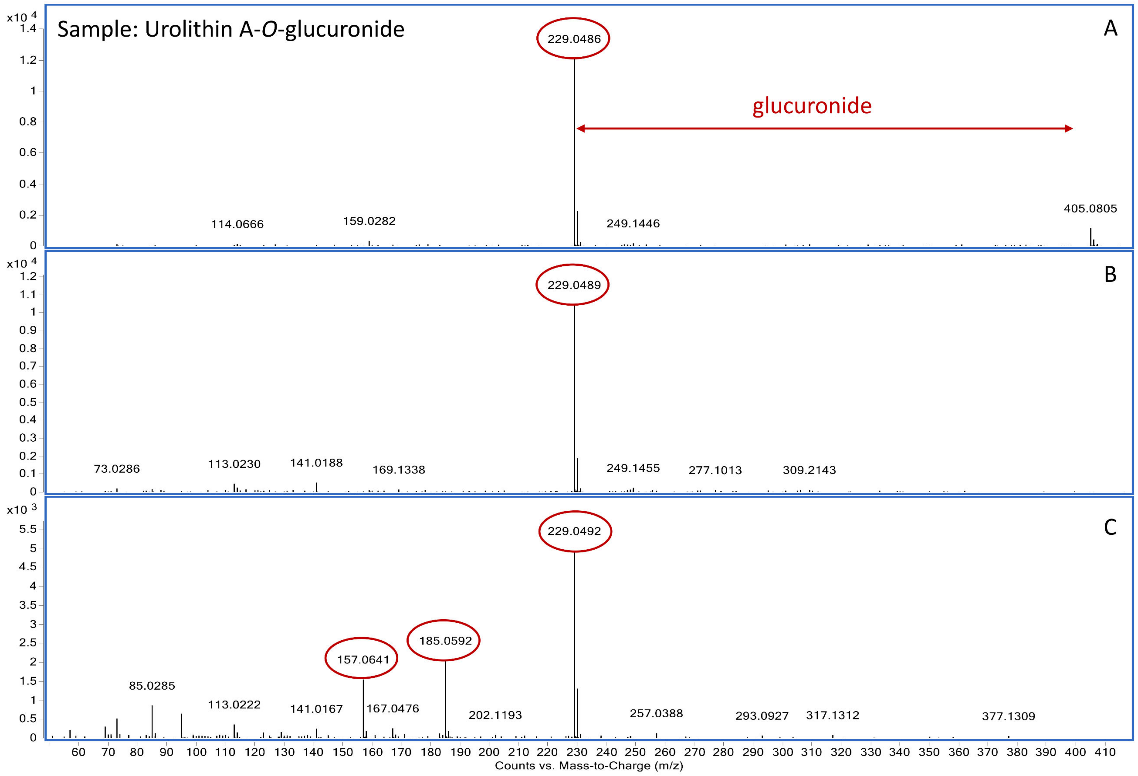

Figure S8. Identification of M19 as urolithin A and M21 as urolithin A-3-*O*-glucuronide by the authentic standard of urolithin A in positive mode. A, B, and C represent the collision energy of 10, 20, and 40 eV respectively on selected precursors. The red circles indicate identical feature fragments in both the urine sample and the standard.

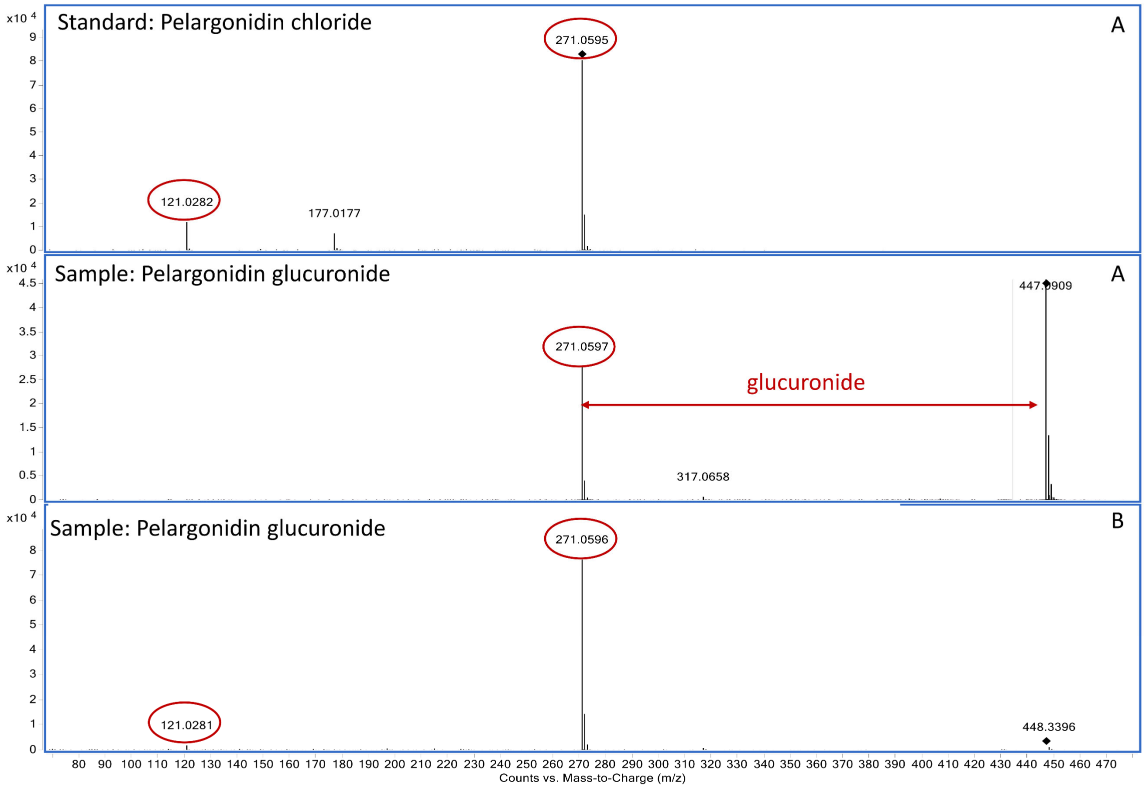

Figure S9. Identification of M16 as pelargonidin glucuronide by the authentic standard of pelargonidin chloride in positive mode. A and B represent the collision energy of 10 and 40 eV respectively on selected precursors. The red circles indicate identical feature fragments in both the urine sample and the standard.

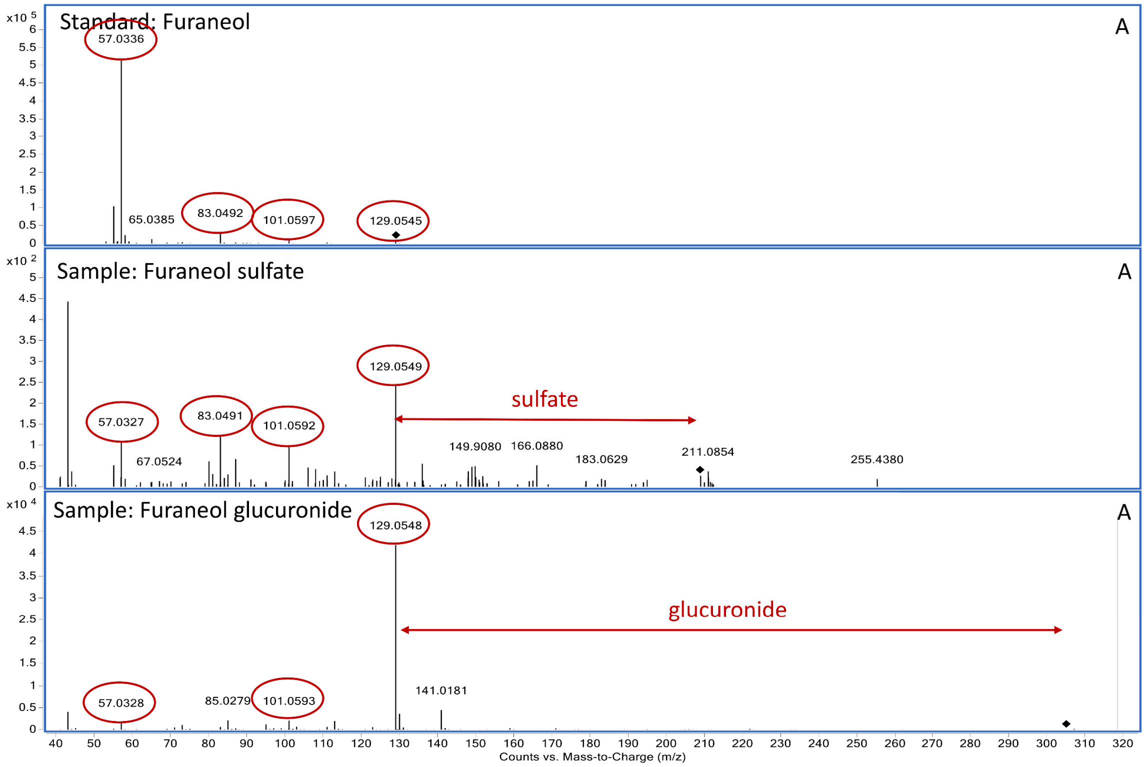

Figure S10. Identification of M17 and M20 as furaneol sulfate and furaneol glucuronide by authentic standard furaneol in positive mode. A represents the collision energy of 20 eV on selected precursors. The red circles indicate identical feature fragments in both the urine sample and the standard.

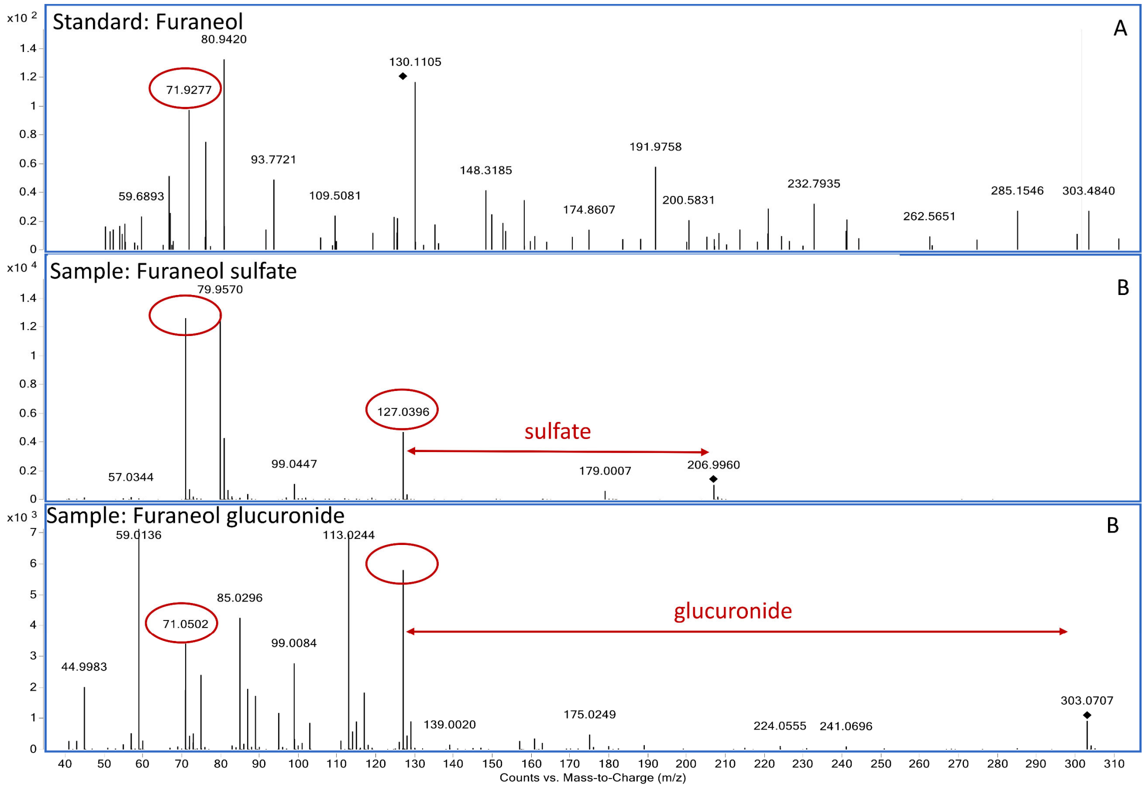

Figure S11. Identification of biomarkers as furaneol sulfate and furaneol glucuronide by authentic standard furaneol in negative mode. A and B represent the collision energy of 55 eV and 20 eV on selected precursors. The red circles indicate identical feature fragments in both the urine sample and the standard.

Table S2. 7 biomarkers with significant differences between each of the three portions

M	RT	Mass	Prec. m/z	Ion	MS/MS	Suggested Metabolite	Formula
M01	1.211	263.9936	262.9866	$[M-H]^-$	79.9564, 96.9603, 167.0204, 183.0290	Methylgallic acid-O-sulfate II	$C_8H_8O_8S$
M02	1.464	237.978	236.9705	$[M-H]^-$	41.0031, 55.0194, 157.0132	Zymonic acid sulfate II	$C_6H_6O_8S$
M03	13.043	456.1618	455.1543	$[M-H]^-$	113.0245, 175.0231, 217.1220, 279.1213	Hydroxy-abscisic acid glucuronide II	$C_{21}H_{28}O_{11}$
M08	4.381	264.0295	263.0219	$[M-H]^-$	168.0429, 183.0656	3-Methoxy-4-hydroxyphenylethyleneglycol sulfate II	$C_9H_{12}O_7S$
M11	6.449	251.9936	250.9859	$[M-H]^-$	79.9568, 171.0292	3-Dehydroshikimate sulfate II	$C_7H_8O_8S$
M12	8.101	374.0839	373.0764	$[M-H]^-$	113.0245, 197.0447	Unknown glucuronide III	$C_{15}H_{18}O_{11}$
M21	13.484	404.0733	405.0808	$[M+H]^+$	229.0512	Urolithin A-3-*O*-glucuronide I	$C_{19}H_{16}O_{10}$

Roman numerals indicate the level of identification, I represents level I identifications, II represents level II identifications, III represents level III identifications.
